# The effect of declining exposure on T cell-mediated immunity to *Plasmodium falciparum –* an epidemiological “natural experiment”

**DOI:** 10.1186/s12916-016-0683-6

**Published:** 2016-09-22

**Authors:** Yaw Bediako, Joyce Mwongeli Ngoi, George Nyangweso, Juliana Wambua, Michael Opiyo, Eunice Wambui Nduati, Philip Bejon, Kevin Marsh, Francis Maina Ndungu

**Affiliations:** 1 Kenya Medical Research Institute, Centre for Geographical Medical Research (Coast), Box 230, 80108 Kilifi, Kenya; 2 Nuffield Department of Clinical Medicine, John Radcliffe Hospital, University of Oxford, Oxford, OX3 9DU UK; 3 The Francis Crick Institute, London, NW7 1AA UK

**Keywords:** Malaria, T cells, Immunity

## Abstract

**Background:**

Naturally acquired immunity to malaria may be lost with lack of exposure. Recent heterogeneous reductions in transmission in parts of Africa mean that large populations of previously protected people may lose their immunity while remaining at risk of infection.

**Methods:**

Using two ethnically similar long-term cohorts of children with historically similar levels of exposure to *Plasmodium falciparum* who now experience very different levels of exposure, we assessed the effect of decreased parasite exposure on antimalarial immunity. Peripheral blood mononuclear cells (PBMCs) from children in each cohort were stimulated with *P. falciparum* and their *P. falciparum*-specific proliferative and cytokine responses were compared.

**Results:**

We demonstrate that, while *P. falciparum*-specific CD4^+^ T cells are maintained in the absence of exposure, the proliferative capacity of these cells is altered considerably. *P. falciparum*-specific CD4^+^ T cells isolated from children previously exposed, but now living in an area of minimal exposure (“historically exposed”) proliferate significantly more upon stimulation than cells isolated from children continually exposed to the parasite. Similarly, PBMCs from historically exposed children expressed higher levels of pro-inflammatory cytokines and lower levels of anti-inflammatory cytokines after stimulation with *P. falciparum*. Notably, we found a significant positive association between duration since last febrile episode and *P. falciparum*-specific CD4^+^ T cell proliferation, with more recent febrile episodes associated with lower proliferation.

**Conclusion:**

Considered in the context of existing knowledge, these data suggest a model explaining how immunity is lost in absence of continuing exposure to *P. falciparum.*

**Electronic supplementary material:**

The online version of this article (doi:10.1186/s12916-016-0683-6) contains supplementary material, which is available to authorized users.

## Background

Malaria remains a significant threat to human health. While it is difficult to estimate attributable numbers of cases and deaths, the recent WHO report suggests at least 198 million cases were reported in 2013, leading to approximately 584,000 deaths [1]. A large proportion of this burden is borne by Africa, where despite massive investments in malaria control and prevention, 57 % of the population continue to live in areas of moderate to intense malaria transmission [2].

Studies in malaria endemic areas consistently demonstrate that incidence and severity of disease decreases significantly with age, indicating that individuals living in these areas acquire a degree of immunity to clinical malaria. This protection is, however, only acquired following repeated infections and is not sterile [3, 4]. Although the mechanisms that underpin naturally acquired immunity to malaria (NAI) remain poorly understood, a review of literature suggests that it is comprised of two main components, namely (1) an anti-parasite component resulting in control of parasite replication and parasite clearance [5, 6] and (2) the ability to tolerate parasites asymptomatically. The latter component is likely to include an immunoregulatory (immune tolerance) element that contributes to control of symptoms and clinical immunity [7, 8]. Antibody-dependent mechanisms play a major role in parasite control and clearance [5], with contributions from innate and cellular arms of immunity [6]. In contrast, the mechanism for the establishment of *Plasmodium falciparum*-specific immune “tolerance” is less understood. Recent studies suggest that repeated exposure to *P. falciparum*, as experienced in areas of high malaria endemicity, is required for the establishment of tolerance [7], which may be associated with the loss and/or altered function of several immune cell types, including γδ T cells [8], αβ T cells [9–12], B cells [13], and myeloid cells [14]. Repeated exposure also results in an expansion of “self-regulating” Th1 cells [7, 15, 16], which produce IL-10 in combination with IFN-γ. IL-10 is a key immunomodulatory cytokine and plays an important role in mouse models of malaria [17–19]. Continual exposure to *P. falciparum* therefore appears to result in modulation of inflammation and associated immunopathology through regulation at multiple levels of the immune system.

Unfortunately, individuals who acquire immunity in malaria endemic areas but then migrate to malaria-free regions for prolonged periods appear to be susceptible to clinical disease upon returning to the endemic region [20–22]. This loss of immune protection may reflect defective antimalarial immunity related to poor induction and maintenance of long-lived memory responses [22]. However, while protective plasma antibody levels decay rapidly (especially in young children) [23, 24], rapid boosting of antibody responses to a number of *P. falciparum* antigens have been reported upon re-exposure following periods of no exposure [25, 26]. In agreement with this, we have previously demonstrated that, while *P. falciparum*-specific antibody levels fall to undetectable levels in the absence of persistent *P. falciparum* exposure, *P. falciparum*-specific memory B cells are maintained at similar levels to those found in children continually exposed to the parasite [27]. Furthermore, one might also infer a loss of tolerance to parasitemia from observations of the cytokine profiles of previously immune, returning travelers [28] and from the fact that these individuals appear to become unwell at parasite densities that they might previously have tolerated asymptomatically [29].

The mechanism(s) by which *P. falciparum*-specific T cell memory is induced and maintained is poorly understood. While some reports have described impairments in the establishment of *P. falciparum*-specific T cell memory (stemming from antigenic diversity [30], infection-related depletion of antigen-specific T cells [31, 32], and impaired dendritic cells [33]), animal models suggest that *P. falciparum*-specific memory T cell populations are maintained normally after infection. To date, only one study has directly assessed the longevity of *P. falciparum*-specific T cell responses in humans following a period of minimal exposure. This study measured *P. falciparum*-specific responses in adults living in an area of low malaria endemicity in Northern Thailand and demonstrated that, while some antimalarial T cell responses (IFN-γ producing T cells) were relatively short-lived, others (IL-10 producing T cells) were maintained for much longer in the absence of exposure [34]. The limitation of this study, however, was that the majority of the malaria-exposed individuals had experienced only one documented episode of malaria in their lifetime. Given the previously described role that endemic malaria has in shaping the *P. falciparum*-specific T cell response [8–12], it is reasonable to suspect that the longevity of memory responses established in individuals previously living in malaria endemic regions (and continually exposed to *P. falciparum*) may differ significantly from those established in individuals only infrequently exposed to malaria.

In this study, we determined the effect of diminished exposure on the size and function of *P. falciparum*-specific T cell memory responses by studying a unique epidemiological “natural experiment” on the coast of Kenya. Here, two ethnically and culturally similar cohorts of children with historically endemic exposure to *P. falciparum* now experience very different levels of exposure. While one group of children has remained continually exposed over the past 8 years (“continually exposed” cohort in Junju), the other has experienced a dramatic reduction in malaria transmission such that exposure has been minimal for over 8 years prior to sampling (“historically exposed” cohort in Ngerenya). By performing a detailed functional characterization of *P. falciparum*-specific T cell responses found in these two otherwise similar groups of children, we hoped to gain insight into the fate of *P. falciparum*-specific T cell immunity as exposure to *P. falciparum* declines. We demonstrate that lack of continuing exposure in the historically exposed cohort resulted in increased levels of *P. falciparum*-specific CD4^+^ T cell proliferation and pro-inflammatory cytokine production.

## Methods

### Study site

The study took place at the KEMRI-Wellcome Trust Research Programme (KWTRP) situated at the Kilifi County Hospital, Kilifi, Kenya. The hospital serves approximately 500,000 people living in Kilifi County. The children investigated were residents of two villages, located within 20 km of each other, with Junju lying on the southern side and Ngerenya on the northern side of an Indian Ocean creek inhabited predominantly by Mijikenda people.

### Study population

Peripheral blood mononuclear cell (PBMC) samples were collected as part of ongoing surveillance of children enrolled in two cohorts at KWTRP in Kilifi, Kenya. Over the last 15 years, there has been a gradual, heterogeneous decline in malaria transmission in Kilifi County [35, 36], whereby Junju village remains stably endemic with two high transmission seasons (May to August and October to December) and a parasite prevalence of 30 % [37, 38] during the dry season, while in Ngerenya (20 km North), parasite prevalence has dramatically reduced from endemic levels of 40 % in 1998, to less than 1 % since 2005 [39]. Children are recruited into the cohorts at birth and actively monitored on a weekly basis for detection of malaria episodes until 15 years of age. Extensive and detailed records of the number and dates of malaria episodes for each child over the period they are enrolled in the cohort are maintained. For this analysis, historically exposed children were selected from Ngerenya on the basis of having confirmed prior exposure to malaria (at least one clinical episode) and matched to continually exposed children of similar age in Junju (Table 1).

**Table 1:** Characteristics of study cohorts

Variable	Ngerenya	Junju
Total number	44	44
Sex (%)		
Male	26 (59)	18 (41)
Female	20 (45)	24 (55)
Age (years)		
Mean (95 % CI)	11.09 (10.66–11.51)	11.16 (10.75–11.57)
Range	7.16–12.94	7.35–12.99
Total no. of previous *P. falciparum* episodes		
Mean (95 % CI)	2.64 (2.01–3.26)	4.48 (3.35–5.60)
Range	1–9	1–16
Time since last episode (mo)		
Median (IQR)	105.8 (78.17–112.5)	12.1 (4.2–36.67)
Range	3.23–143.6	2.03–83.1
*P. falciparum* infection status at sampling (% positive)		
By blood smear	1 (2.2)	11 (25)
By PCR	1 (2.2)	15 (34)

### Sample collection and preparation

Venous blood (5 mL; for immunological studies) and blood smears (for detection and subsequent calculation of *P. falciparum* parasitemia) were collected from each participant in a preseason cross-sectional survey in May 2012, a time preceded by 4 months of minimal *P. falciparum* transmission in Junju. PBMCs were isolated by density gradient centrifugation (Ficoll-Histopague, GE Life Sciences) and stored in liquid nitrogen till the assays were performed.

### Determination of parasitemia

Thick and thin blood smears were stained with Giemsa and *P. falciparum*-infected red cells counted against 500 leukocytes and 1000 red blood cells, respectively. To further confirm that previously exposed children were uninfected, a *P. falciparum*-specific PCR was performed, as previously described [40].

### Malaria antigen

*P. falciparum* blood-stage parasites (laboratory-adapted local field isolate) were grown by standard methods and harvested at 5–10 % parasitemia. Red blood cells infected (iRBC) with trophozoite stage parasites were purified via density gradient centrifugation using Percoll (GE, Life Sciences) washed and cryopreserved in glycerolyte. A single batch of parasites was used throughout the study. Aliquots of this batch were stored in liquid nitrogen until required. Uninfected red blood cells (uRBC) were prepared and stored in a similar manner for use as controls.

### Intracellular cytokine staining

Thawed PBMCs were rested overnight in media supplemented with 10 % fetal bovine serum (Gibco) and restimulated with media, uRBCs, iRBCs, and plate-bound anti-CD3 (BioLegend) at 7.5 × 10^5^ cells per condition. An effector-to-target ratio of 1:3 was used with uRBCs and iRBCs. Anti-CD28 and anti-CD49d were added for co-stimulation (3 μg/mL BioLegend). Brefeldin A (BD Pharmingen) was added at 6 hours of incubation at a final concentration of 10 μg/mL to inhibit cytokine secretion. At 24 hours, cells were washed, surface stained, fixed, permeabilized, and stained for intracellular cytokines per standard protocols (BD Pharmingen).

Surface and intracellular staining of PBMCs was done with standard staining protocols using the following antibodies: fluorescein isothiocyanate (FITC)-conjugated anti-CD45RO, allophycocyanin (APC)-Cy7-conjugated anti-CD3, Brilliant Violet 421-conjugated anti-IL-10 (panel 1), Brilliant Violet 421-conjugated anti-TNF-α (panel 2), phycoerythrin (PE)-conjugated anti-IL-4 (panel 2), APC-conjugated anti-CCR7 (BioLegend), PerCP-Cy5.5-conjugated anti-CD8, PE-Cy7-conjugated-anti CD27, PE-conjugated anti-IFN-γ (panel 1) (BD Phamingen), and PE-Texas Red-conjugated CD4 (Invitrogen). LIVE/DEAD aqua amine was included to exclude dead cells from downstream analysis (although the malaria specific analysis did not include CD8^+^ T cells, they were stained to facilitate gating on CD4^+^CD8^–^ T cells) (see Additional file 1: Table S1 for complete panel).

### CFSE proliferation assay

Thawed PBMCs were rested overnight in media supplemented with 10 % fetal bovine serum. Cells were washed and 1 × 10^6^ cells were labeled with 1 mL of 5 μM CFSE (BioLegend) following an established protocol reported elsewhere [41]. CFSE-labeled PBMCs were incubated in a 96-well culture plate and stimulated with media, uRBCs, iRBCs, and plate-bound anti-CD3 (BioLegend) at a density of 2.5 × 10^5^ cells per condition. As before, an effector-to-target ratio of 1:3 was used with uRBCs and iRBCs. Anti-CD28 and anti-CD49d were added for costimulation (3 μg/mL BioLegend). At day 7, supernatants were collected and frozen for downstream cytokine analysis and the cells washed and stained with surface antibodies (Brilliant Violet 421-conjugated anti-CD4, APC-conjugated anti-CCR7, APC-Cy7-conjugated anti-CD3 (BioLegend), PE-Cy7-conjugated anti-CD27, and PerCP-Cy5.5-conjugated anti-CD8 (BD Pharmingen)) before acquisition. Once again, LIVE/DEAD aqua amine was included to exclude dead cells from downstream analysis.

### Flow cytometry

Cytokine production by CD4^+^ T cells was analyzed using two panels; panel 1: IFN-γ and IL-10; panel 2: IL-4 and TNF-α (both panels assessed surface expression of a number of markers of CD4^+^ T cell memory phenotype). At least 100,000 lymphocytes were acquired on a 9-color Cyan ADP flow cytometer (Beckman Coulter). Color compensations were performed using samples stained for each of the fluorochromes used. Data were analyzed using FlowJo (Tree Star). Percentages of iRBC-stimulated cytokine producing CD4^+^ T cells are reported after background subtraction of the frequency of the identically gated population of cells from the same sample stimulated with uRBCs and are expressed as a percentage of total CD4^+^ T cells. For the phenotypic analysis of CD4 T cell memory subsets, the population of cells that express each marker within the CD4 T cell population was entered into a ‘Boolean gating’ analysis [42] that separately identifies all the subpopulations expressing each possible combination of markers. The frequency of each specific CD4^+^ T cell subpopulation is expressed as a percentage of CD4^+^ T cells. In experiments with CFSE labeled cells, the percentages of divided CD4^+^ T cells after iRBC stimulation are reported after subtraction of the percentage of divided CD4^+^ T cells after uRBC stimulation.

### Luminex analysis of culture supernatants

Supernatants were thawed and immediately analyzed using a Human Cytokine Magnetic 25-Plex assay (Invitrogen) as recommended by the manufacturer. The following cytokines were measured: IL-1β, IL-1RA, IL-2, IL-2R, IL-4, IL-5, IL-6, IL-7, IL-8, IL-10, IL-12 (p40), IL-13, IL-15, IL-17, TNF-α, INF-α, IFN-γ, GM-CSF, CCL3 (MIP-1α), CCL4 (MIP1β), CXCL10 (IP-10), CXCL9 (MIG), Eotaxin, CCL5 (RANTES), and CCL2 (MCP-1). Briefly, 50 μL of culture supernatant was diluted 1:2 in medium and incubated with anti-cytokine antibody-coupled magnetic beads for 2 hours at room temperature while shaking at 500 rpm in the dark. The beads were then incubated with 100 μL of a biotinylated detector antibody for 1 hour at room temperature, before incubation with streptavidin R-phycoerythrin for 30 mins (between each step, the beads were washed in wash buffer using a magnetic separator). After a final wash, beads were resuspended in 125 μL of buffer and 100 beads counted for each cytokine in a Bio-Plex MAGPIX multiplex reader (Bio-Rad Laboratories, Inc.). Final concentrations were calculated from the mean fluorescence intensity and expressed in pg/mL using standard curves with known concentrations of each cytokine.

### Statistical methods

All statistical analyses were performed using Prism 6.0 (GraphPad). Mann–Whitney U-test and Kruskal–Wallis tests were used to compare continuous variables between two and more than two groups, respectively. Correlations between different continuous measures were determined using Spearman’s rank correlation coefficient. For all tests, two-tailed *P* values were considered significant if *P* < 0.05.

## Results

### *P. falciparum*-specific CD4^+^ T cells are maintained in the absence of continual exposure

**Fig. 1:**
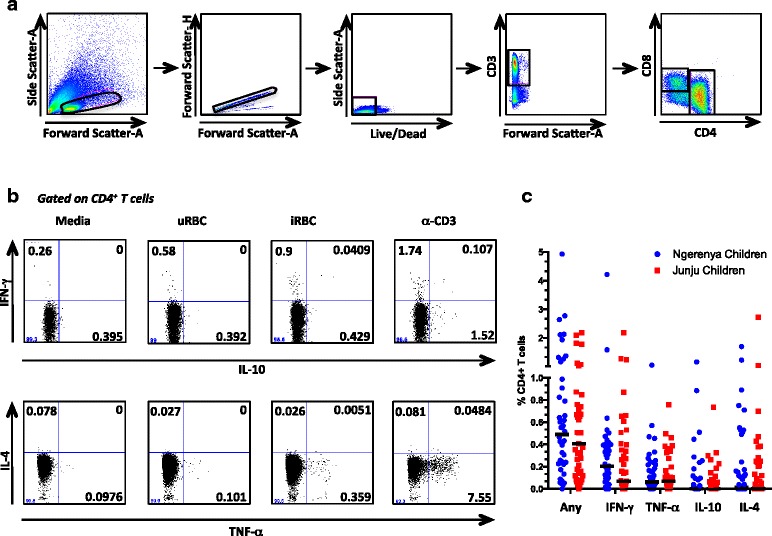
*P. falciparum*-specific CD4^+^ T cells are maintained in the absence of continual exposure to *P. falciparum*. **a** Gating strategy to identify live CD4^+^ T cells. **b** Intracellular cytokine staining assay demonstrating CD4^+^ T cell response of one representative child to stimulation with *P. falciparum*-infected RBCs (iRBC), negative controls: media and uninfected RBC (uRBC) and positive control: plate-bound anti-CD3 (α-CD3). Shown is CD4^+^ T cell production of IFN-γ and IL-10 (upper panel) as well as IL-4 and TNF-α (lower panel). **c** Overall frequency of CD4^+^ T cells producing any cytokine, IFN-γ, TNF-α, IL-10, and IL-4, respectively. *P. falciparum*-specific cytokine production was calculated as percentage of CD4^+^ T cells producing a particular cytokine following iRBC stimulation minus uRBC stimulation. Blue dots represent historically-exposed children living in Ngerenya (*n* = 44), while red squares represent continually exposed children living in Junju (*n* = 44). Horizontal black lines indicated the median response for each group. No statistically significant differences were observed between historically and continually exposed children, as assessed by Mann–Whitney U test

To determine whether malaria-specific CD4^+^ T cells are maintained in the absence of continued exposure, we compared the frequency and function of *P. falciparum*-specific CD4^+^ T cell responses between historically and continually exposed children. PBMC from each group of children were stimulated with *P. falciparum*-iRBC and analyzed by flow cytometry for production of IFN-γ, TNF-α, IL-10, and IL-4 (Fig. 1a, b). There was no significant difference between the proportions of CD4^+^ T cells producing each cytokine between the two groups of children (IFN-γ *P* = 0.26, TNF-α *P* = 0.65, IL-10 *P* = 0.81, IL-4 *P* = 0.66), suggesting that maintenance of *P. falciparum*-specific CD4^+^ T cells is not dependent on continual exposure to the parasite (Fig. 1c). In fact, there appeared to be a trend (though not statistically significant) towards higher responses (IFN-γ and any cytokine) in the Ngerenya cohort (i.e. historically exposed children). It is important to note that, similar to the findings of a study of a cohort of children from the same area [43], the majority of children sampled from both groups had undetectable levels of IFN-γ/IL-10 co-producing CD4^+^ T cells.

### Overall memory phenotype of *P. falciparum*-specific CD4^+^ T cells remains unchanged in the absence of continual exposure

**Fig. 2:**
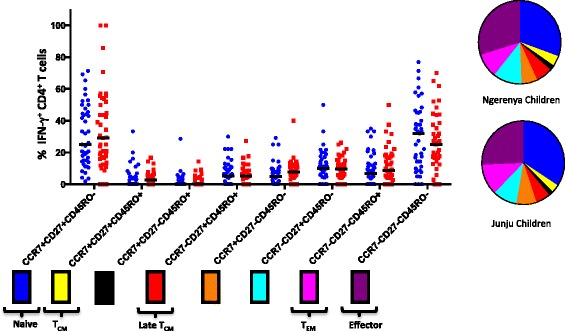
Memory phenotype of *P. falciparum*-specific CD4 ^+^ T cell population maintained in absence of continual exposure. Differential expression of CCR7, CD27, and CD45RO by *P. falciparum*-specific CD4^+^ T cells (producing IFN-γ upon stimulation with *P. falciparum*-infected red blood cells). Each phenotype (defined by a specific combination of markers) is shown beneath the X-axis. Where a specific phenotype has been ascribed to a particular differentiation stage, this is specified. T_CM_ and T_EM_ refer to central memory and effector memory cell populations, respectively. The frequency of each subset is expressed as a percentage of the total IFN-γ^+^ CD4^+^ T cell population and was compared between historically exposed children (Ngerenya) and continually exposed children (Junju). Horizontal black lines represent median responses in each group. Mann–Whitney U-test was used for pairwise analysis of differences between groups – no significant differences were observed. Pie charts illustrate relative proportions of each of the combinations of markers in the two groups of children

Persistent exposure to infection can result in significant remodeling of the T cell population, changing the relative frequencies of different memory subsets with potentially significant immunological consequences [44, 45]. Even subtle differences in environmentally driven T cell activation can result in phenotypic and functional changes to the T cell compartment [46]. To assess whether the phenotype of *P. falciparum*-specific CD4^+^ T cells changed with declining exposure to *P. falciparum*, we compared the surface phenotype of *P. falciparum*-specific CD4^+^ T cells (producing IFN-γ in response to *P. falciparum*) isolated from each group of children (Fig. 2). Once again, we did not observe any significant differences between the two groups for any of the T cell memory subsets we examined. This would suggest that not only is the size of the *P. falciparum*-specific CD4^+^ T cell population maintained in the absence of exposure, but the overall memory differentiation profile of the population is also maintained.

### *P. falciparum*-specific T cell proliferation is significantly enhanced after period of minimal exposure

**Fig. 3:**
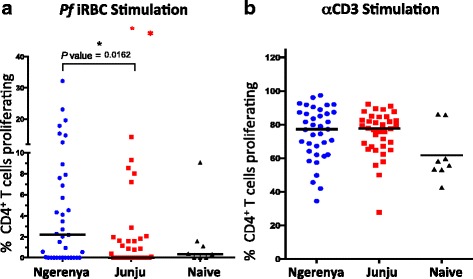
*P. falciparum*-specific CD4^+^ T cell proliferation enhanced after period of minimal exposure. Proportion of *P. falciparum*-specific CD4^+^ T cells proliferating in response to iRBC stimulation is significantly higher in children with little current exposure to malaria (Blue dots, Ngerenya, *n* = 37) compared to continually exposed children (Red squares, Junju, *n* = 37) and malaria-naïve children (Black triangles, *n* = 8). *P. falciparum*-specific proliferation was calculated as percentage of CFSE-lo CD4^+^ T cells following iRBC stimulation minus uRBC stimulation. Non-specific CD4^+^ T cell proliferation in response to plate-bound anti-CD3 stimulation was also compared across the three groups. Statistically significant *P* values (*P* < 0.05) are indicated by asterisk (in red for Kruskal–Wallis or black for Mann–Whitney U-tests, respectively)

*P. falciparum*-specific T cell proliferation has been shown to be impaired in heavily exposed children [8, 15]. While the frequency and memory phenotype of *P. falciparum*-specific CD4^+^ T cells appear to be unaffected by a decline in exposure, we were interested in whether *P. falciparum*-specific proliferative responses would differ based on levels of recent exposure. We compared proliferation of *P. falciparum*-specific CD4^+^ T cells between historically (*n* = 37) and continually (*n* = 37) exposed children following stimulation with iRBC. Prior reports [34] have described non-specific proliferation of T cells in response to *Pf* antigens. Therefore, we also included samples from malaria-naïve (based on weekly active surveillance since enrollment into cohort at or soon after birth) children of similar age (*n* = 8). *P. falciparum*-specific CD4^+^ T cell proliferation was significantly higher in historically exposed children than in those who have been continually exposed (*P* = 0.016) (Fig. 3a). In keeping with previous reports of exposure dependent impairment of T cell proliferation [47–49], *P. falciparum*-specific CD4^+^ T cell proliferation in continually exposed children was not significantly different from the level of proliferation observed in malaria-naïve children. When we compared the proliferation of CD4^+^ T cells between the three groups after non-specific stimulation with plate-bound anti-CD3 (Fig. 3b), CD4^+^ T cells from all three groups proliferated to similar levels confirming that the difference in proliferation was unique to *P. falciparum*-specific T cells and not representative of widespread suppression of the whole CD4^+^ T cell population. This result suggests that *P. falciparum*-specific CD4^+^ T cell proliferative impairment is dependent on continued exposure to the parasite and is lost after a period of minimal exposure.

### *P. falciparum*-inducible pro-inflammatory cytokines are upregulated in historically exposed relative to continually exposed children

**Fig. 4:**
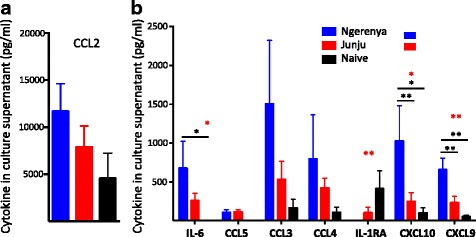
*P. falciparum*- induced pro-inflammatory cytokine production enhanced after period of minimal exposure. Peripheral blood mononuclear cells isolated from children with little current exposure to malaria (Blue bars, Ngerenya, n = 15), continually exposed children (Red bars, Junju, n = 15), and malaria-naïve children (Black bars, *n* = 5) were stimulated for 7 days with *P. falciparum*-infected or uninfected red blood cells (iRBC or uRBC). Culture supernatants were collected and analyzed using a multiplex assay for 25 cytokines. *P. falciparum*-specific cytokine production was calculated as concentration of cytokine following iRBC stimulation minus uRBC stimulation. Results are shown for cytokines which were detected at a net concentration ≥ 40 pg/mL. **a** Concentration of *P. falciparum*-induced CCL2 in each group. **b** Concentration of *P. falciparum*-specific IL-6, CCL5, CCL3, CCL4, IL-1RA, CXCL10, and CXCL9 in each group. Bars indicate mean cytokine concentration (pg/mL) with standard error of the mean (SEM) also indicated. Statistically significant differences (*P < 0.05, **P < 0.01) are indicated by asterisk (in red for Kruskal–Wallis or black for Mann–Whitney U-tests, respectively)

In addition to inhibiting *P. falciparum*-specific T cell proliferation, repeat exposure to malaria has been shown to downregulate the expression of several cytokines associated with malaria-inducible inflammation [7]. To assess whether the enhanced CD4^+^ T cell proliferation we observed in historically exposed children was indicative of an enhanced malaria-inducible inflammatory response, we compared *P. falciparum*-inducible cytokine levels in supernatants from a subset of iRBC-stimulated PBMCs from each group using a multiplex assay. Of the 25 cytokines assayed, eight (CCL2, IL-6, CCL5, CCL3, CCL4, IL-1RA, CXCL10, and CXCL9) were expressed at levels above a quantifiable limit of 40 pg/mL. While CCL2 was the most abundant *P. falciparum*-inducible cytokine, there was no significant difference in its expression between historically and continually exposed children (Fig. 4a). We did, however, find that expression of the pro-inflammatory cytokines, IL-6, CXCL9, and CXCL10 were significantly higher in historically exposed than continually exposed children (IL-6 *P* <0.05, CXCL9 *P* <0.01, CXCL10 *P* <0.01), while the anti-inflammatory cytokine IL-1RA was downregulated in historically exposed children relative to continually exposed children (Fig. 4b). A number of other cytokines (IL-4, IL-13, IL-10, IL-15, IL-5, IFN-γ, and IL-17) were detected at levels too low to be precisely quantified by standard curve; however, we were able to compare their relative levels between groups by comparing the mean fluorescence intensities detected. In agreement with the profile exhibited by the more highly expressed cytokines, we found significantly higher levels of the pro-inflammatory cytokines, IFN-γ, IL-5, IL-13, IL-15, and IL-17 in historically exposed than continually exposed children (Additional file 2: Figure S1). These results would suggest that the mechanism(s) responsible for restraining *P. falciparum*-inducible inflammation observed in highly exposed individuals is lost following a period of minimal exposure, resulting in a more pronounced inflammatory response upon re-exposure.

### *P. falciparum*-specific CD4^+^ T cell proliferation correlates with recent clinical malaria in continually exposed children

**Fig. 5:**
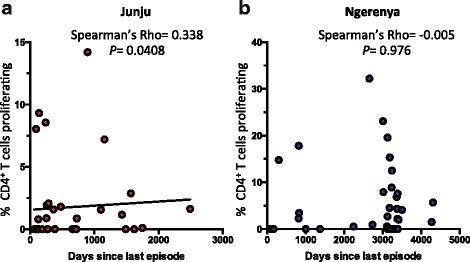
Variations in T cell proliferation can be explained by duration since last episode in continually exposed children but not historically exposed children. **a** Proliferation of *P. falciparum*-specific CD4^+^ T cells is positively associated with days since last clinical malaria episode in continually exposed children (Junju) Spearman’s *Rho* = 0.338, *P* = 0.0408. **b** *P. falciparum*-specific CD4^+^ T cell proliferation does not correlate with days since last clinical malaria episode in children with little current exposure (Ngerenya) Spearman’s *Rho* = -0.005, *P* = 0.976

Since it has previously been demonstrated that there is a strong relationship between *P. falciparum*-specific T cell function and time elapsed since last malaria episode [15], we were interested in determining whether this relationship is maintained after a period of minimal exposure. We observed a strong positive correlation between *P. falciparum*-specific CD4^+^ T cell proliferation and the duration since last clinical episode of malaria (Spearman’s *Rho* = 0.34, *P* = 0.04) in continually exposed children (Fig. 5a), with more recent malaria associated with lower CD4^+^ T cell proliferation. However, no statistically significant association was observed between *P. falciparum*-specific CD4^+^ T cell proliferation and duration since last clinical malaria episode (Spearman’s *Rho* = –0.005, *P* = 0.98) in historically exposed children (Fig. 5b). While all the children were asymptomatic at blood draw, approximately one third of continually exposed children were determined to be parasite positive by PCR. Interestingly, we did not find an association between asymptomatic parasitemia and CD4^+^ T cell proliferation (Additional file 3: Figure S2). Variations in *P. falciparum*-specific CD4^+^ T cell proliferation can thus be explained by duration since last clinical episode in continually exposed children but not historically exposed children, providing further evidence that *P. falciparum*-specific CD4^+^ T cell proliferative impairment is lost in the absence of continued exposure.

## Discussion

In this study, we sought to determine the effect that declining exposure to *P. falciparum* has on previously acquired *P. falciparum*-specific T cell immunity by comparing two cohorts of otherwise similar children with different levels of current exposure. We demonstrate that, while *P. falciparum*-specific CD4^+^ T cells are maintained at similar levels and with similar memory phenotype after a significant period (median: 8.7 years; interquartile range: 4.1–9.1 years) of minimal exposure to the parasite*,* the proliferative capacity of these cells appeared to be altered considerably. In keeping with previous reports [8, 15], we found evidence of significantly impaired CD4^+^ T cell proliferation in continually exposed children, with levels of *P. falciparum*-specific CD4^+^ T cell proliferation in these children indistinguishable from the levels in malaria-naïve children. In contrast, after a period of minimal exposure, *P. falciparum*-specific CD4^+^ T cells proliferated robustly upon re-stimulation in vitro, suggesting that the regulatory mechanism responsible for inhibiting *P. falciparum*-specific CD4^+^ T cell proliferation is dependent on exposure to the parasite. Importantly, this exposure-dependent inhibition of CD4^+^ T cell proliferation was restricted to *P. falciparum*-specific CD4^+^ T cells, since the levels of CD4^+^ T cell proliferation in response to anti-CD3 stimulation were similar across the three groups.

While we did not find an association between asymptomatic parasitemia and CD4^+^ T cell proliferation, we did find a significant positive association between duration since last febrile episode and *P. falciparum*-specific CD4^+^ T cell proliferation (in continually exposed children), with more recent febrile episodes associated with lower proliferation. This result is in agreement with a recent study of seasonal malaria in Mali, where successive exposure to *P. falciparum* resulted in downregulation of pro-inflammatory responses and an upregulation of cytokines responsible for control of inflammation [7]. While that study did not assess *P. falciparum*-specific CD4^+^ T cell proliferation, our finding that PBMCs from historically exposed children express higher levels of pro-inflammatory cytokines (including IL-6, IL-5, CXCL9, and CXCL10), but lower levels of anti-inflammatory cytokines (including IL-1RA) than continually exposed children following re-stimulation with *P. falciparum* suggests that impaired *P. falciparum*-specific CD4^+^ T cell proliferation is a further reflection of malaria-induced immunoregulation. Interestingly, IL-6, CXCL9, and CXCL10 have all been demonstrated to stimulate T cell proliferation [50–52], with IL-6 in particular also promoting T cell survival and inhibiting activation-induced cell death [53]. Furthermore, IL-1RA, which we found to be elevated in continually exposed children relative to historically exposed children, has been found to inhibit T cell responses to antigenic stimulation [54]. We acknowledge the findings of a recent study reporting a decline in malaria antigen specific- IFN-γ, IL-10, and TNF-α responses in individuals following a period of low exposure; however, the profile of cytokine responses reported in that study varied substantially by antigen and the reported decline in cytokine levels was not always maintained beyond 6 months of follow-up. Importantly, that study measured cytokine responses to individual peptide antigens, while we measured the response to the whole parasite. The gap in exposure reported was also significantly shorter than in our study, suggesting that, while there might be antigen-specific fluctuations in cytokine levels over short periods of low exposure, the cytokine response profile after longer gaps in exposure may be altered significantly.

Our results provide clear evidence that (1) *P. falciparum*-specific CD4^+^ T cells are maintained in the absence of continual exposure to the parasite, (2) continual exposure to *P. falciparum* induces a strong immunoregulatory response capable of dampening infection-associated inflammation, and (3) *P. falciparum*-specific CD4^+^ T cell proliferation (following in vitro stimulation) is significantly enhanced after a period of minimal exposure. While future studies will be needed to precisely define these mechanisms, our data suggests that the mechanisms responsible for mediating malaria-induced immunoregulation (potentially critical for NAI) could be lost in the absence of continual exposure to the parasite. Such mechanisms are likely to involve regulatory T cells [55, 56] and atypical/exhausted lymphocytes [9, 10] that have been shown to expand with continuous exposure to malaria.

As we have mentioned previously, immune individuals who migrate to malaria-free regions for prolonged periods may become susceptible to clinical disease upon re-exposure, but still demonstrate less severe outcomes than malaria-naïve individuals [20–22]. Such individuals mount robust inflammatory responses to relatively few parasites [21, 57], suggesting that they retain some ability to control parasite replication but are unable to modulate malaria-induced inflammation [22]. Considered in the context of our data and existing knowledge about how malaria infection modulates several components of the immune system, these epidemiologic observations suggest a model by which clinical immunity is lost in absence of exposure to *P. falciparum*. We hypothesize that malaria-naïve individuals who become infected with *P. falciparum* are unable to control parasite growth resulting in inflammation and associated acute febrile illness. Continually exposed individuals are able to restrict parasite growth (mediated in large part by antibody-dependent mechanisms) and also able to modulate parasite-induced inflammation and T cell proliferation, allowing them to remain afebrile despite the persistence of low level parasitemia. Historically exposed individuals, on the other hand, are capable of limiting parasite growth but have lost the ability to modulate parasite-induced inflammation, resulting in an exaggerated inflammatory reaction and acute febrile illness in response to relatively few parasites. The precise mechanism of malaria-induced immunoregulation remains to be investigated and likely involves both innate and adaptive components. Future work will need to assess the possible contributions of “malaria toxin”-induced TLR hypo-responsiveness [58], as well as more T cell intrinsic changes that may explain the observed loss of clinical immunity with lack of exposure. One significant caveat of this study is that we are unable to determine whether the immunoregulation observed in historically exposed children represents a decline of pre-established immunoregulatory mechanisms or a failure to adequately develop these mechanisms under conditions of declining exposure. Longitudinal cohort studies and controlled human challenge experiments in naturally immune populations will be needed to fully investigate this question.

## Conclusions

The last decade has seen vast investments in malaria control and the associated decline in transmission is reason to be encouraged. However, the heterogeneous nature of this decline [2] may leave large populations of previously protected individuals susceptible to clinical disease. This is particularly important if a population of children emerge who have lost the ability to control the inflammatory response to malaria and are therefore at higher risk of illness. Furthermore, such pre-existing immune regulation may be responsible for the observed reductions in immunogenicity for malaria experimental vaccines in malaria-exposed populations relative to malaria naïve ones [37]. Understanding the mechanisms of *P. falciparum*-induced immunoregulation, the role that this plays in NAI, and how immunity is affected by a decline in exposure will be critical in the design and implementation of an effective vaccine, which remains the best long-term preventative measure against malaria.

## Additional files

Additional file 1: Table S1. Flow cytometry staining panels﻿. (XLSX 8 kb) Additional file 2: Figure S1. Relative amounts of *P. falciparum*-induced cytokines in historically and continually exposed children. Peripheral blood mononuclear cells were isolated from children with little current exposure to malaria (Blue bars, Ngerenya, *n* = 15) compared to continually exposed children (Red bars, Junju, *n* = 15) and malaria-naïve children (Black bars, *n* = 5). Culture supernatants were analyzed using a multiplex assay for 25 cytokines. *P. falciparum*-specific cytokine production was calculated as mean fluorescent intensity (MFI) of following *P. falciparum*-infected red blood cell stimulation minus uninfected red blood cell stimulation. Results are shown for cytokines that were detected at levels that precluded precise quantification by standard curve. Bars indicate MFI with standard error of the mean (SEM) also indicated. Statistically significant differences (**P* < 0.05, ***P* < 0.01) are indicated by asterisk (in red for Kruskal–Wallis or black for Mann–Whitney U-tests, respectively). (PDF 67 kb) Additional file 3: Figure S2. Proliferation of *P. falciparum*-specific CD4^+^ T cells in continually exposed children is unaffected by asymptomatic parasitemia. Proportion of *P. falciparum*-specific CD4^+^ T cells proliferating in response to *P. falciparum*-infected red blood cells (iRBC) does not differ based on parasite status. *P. falciparum*-specific proliferation in continually exposed children was calculated as percentage of CFSE-lo CD4^+^ T cells following iRBC stimulation minus uRBC stimulation. Children were stratified according to parasite status at blood draw (determined by PCR); red squares represent children who were positive for parasites and black squares represent children who were parasite free. No statistically significant differences were observed as assessed by Mann–Whitney U test. (PDF 42 kb)
