# Supplementary figures and images for: The effect of declining exposure on T cell-mediated immunity to Plasmodium falciparum – an epidemiological “natural experiment”
Source: BMC Med. 2016 Sep 22;14:143. doi: 10.1186/s12916-016-0683-6 (PMC5034532; doi:10.1186/s12916-016-0683-6)

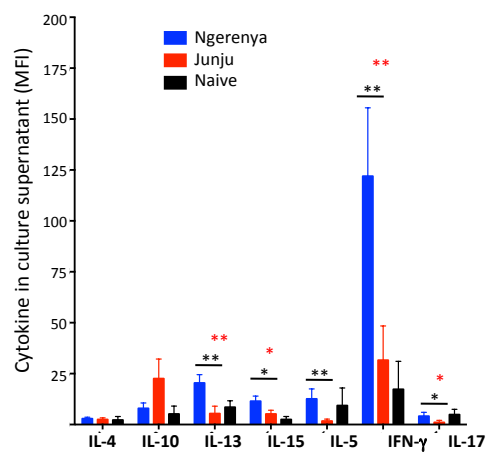

Supplement: Additional file 2: Figure S1. — Relative amounts of P. falciparum-induced cytokines in historically and continually exposed children. Peripheral blood mononuclear cells were isolated from children with little current exposure to malaria (Blue bars, Ngerenya, n = 15) compared to continually exposed children (Red bars, Junju, n = 15) and malaria-naïve children (Black bars, n = 5). Culture supernatants were analyzed using a multiplex assay for 25 cytokines. P. falciparum-specific cytokine production was calculated as mean fluorescent intensity (MFI) of following P. falciparum-infected red blood cell stimulation minus uninfected red blood cell stimulation. Results are shown for cytokines that were detected at levels that precluded precise quantification by standard curve. Bars indicate MFI with standard error of the mean (SEM) also indicated. Statistically significant differences (*P < 0.05, **P < 0.01) are indicated by asterisk (in red for Kruskal–Wallis or black for Mann–Whitney U-tests, respectively). (PDF 67 kb) [file 12916_2016_683_MOESM2_ESM.pdf]

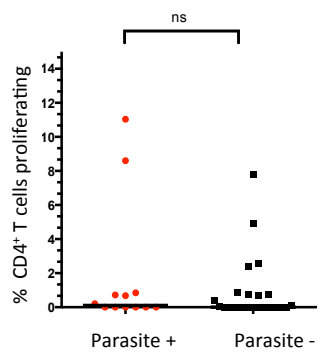

Supplement: Additional file 3: Figure S2. — Proliferation of P. falciparum-specific CD4+ T cells in continually exposed children is unaffected by asymptomatic parasitemia. Proportion of P. falciparum-specific CD4+ T cells proliferating in response to P. falciparum-infected red blood cells (iRBC) does not differ based on parasite status. P. falciparum-specific proliferation in continually exposed children was calculated as percentage of CFSE-lo CD4+ T cells following iRBC stimulation minus uRBC stimulation. Children were stratified according to parasite status at blood draw (determined by PCR); red squares represent children who were positive for parasites and black squares represent children who were parasite free. No statistically significant differences were observed as assessed by Mann–Whitney U test. (PDF 42 kb) [file 12916_2016_683_MOESM3_ESM.pdf]
